# Effect of finerenone on renal function in patients with type 2 diabetic nephropathy: a retrospective cohort study

**DOI:** 10.3389/fendo.2026.1753126

**Published:** 2026-02-10

**Authors:** Xu-Ying Liu, Ya-Min Zhao, Ya-Guang Zhang, Yi Liu, Bo-Ya Wang, Zhen-Zhen Hao, Man-Hui Hu

**Affiliations:** 1 Department of Endocrinology, Affiliated Hospital of Hebei University, Baoding, Hebei, China; 2 Department of Emergency, Baoding First Central Hospital, Baoding, Hebei, China; 3 Department of Nursing, Hebei University, Baoding, Hebei, China; 4 Department of Nephrology, Affiliated Hospital of Hebei University., Baoding, Hebei, China; 5 Department of Gastrointestinal Surgery, Affiliated Hospital of Hebei University, Baoding, Hebei, China

**Keywords:** albuminuria, chronic kidney disease, diabetic nephropathies, finerenone, glomerular filtration rate

## Abstract

**Background:**

Diabetic nephropathy (DN) remains a major cause of chronic kidney disease despite optimized renin–angiotensin system blockade. This study aimed to evaluate the efficacy and safety of adding finerenone to angiotensin-converting enzyme inhibitor/angiotensin II receptor blocker (ACEI/ARB) therapy and to identify predictors of clinically meaningful renal deterioration in patients with DN.

**Methods:**

This retrospective cohort study enrolled adult patients (18–80 years) with a confirmed diagnosis of DN according to American Diabetes Association and Kidney Disease: Improving Global Outcomes criteria, who had complete baseline and follow-up data. Patients with type 1 diabetes, non-diabetic kidney disease, severe hepatic dysfunction, advanced heart failure, recent acute cardiovascular events, or baseline hyperkalemia were excluded. A total of 240 patients treated between January 2019 and December 2024 were included. Patients receiving ACEI/ARB monotherapy (control group, n = 124) were compared with those receiving ACEI/ARB plus finerenone (observation group, n = 116). Renal and metabolic parameters were assessed at baseline and after 24 weeks. Between-group comparisons were performed using appropriate parametric or nonparametric tests, and multivariable logistic regression analysis was conducted to identify independent predictors of a ≥15% estimated glomerular filtration rate (eGFR) decline.

**Results:**

After 24 weeks, patients receiving finerenone showed significantly lower Scr (128.3 ± 27.6 μmol/L vs. 140.8 ± 35.1 μmol/L, P = 0.002), higher eGFR (56.8 ± 11.4 vs. 50.1 ± 12.3 mL/min/1.73 m², P < 0.001), and lower UACR (301.4 ± 142.7 vs. 398.7 ± 176.8 mg/g, P < 0.001) than controls. Finerenone treatment independently protected against renal deterioration (adjusted odds ratio [aOR] = 0.473, 95% CI: 0.253–0.883, P = 0.019), while longer diabetes duration, lower baseline eGFR, and higher UACR predicted ≥15% eGFR decline. Both regimens were well tolerated, with no increase in severe hyperkalemia or serious adverse events.

**Conclusions:**

Adding finerenone to ACEI/ARB therapy improved renal parameters over 24 weeks and was independently associated with reduced risk of clinically meaningful eGFR decline without excess serious adverse events.

## 1 Introduction

Diabetic nephropathy (DN), referred to as diabetic kidney disease, is a major microvascular complication of diabetes mellitus and is clinically defined by persistent albuminuria and/or a progressive decline in estimated glomerular filtration rate (eGFR) attributable to diabetes, in the absence of alternative primary kidney diseases, according to contemporary guideline-based diagnostic frameworks. It represents the leading cause of chronic kidney disease (CKD) and end-stage renal disease worldwide. Its pathogenesis reflects intersecting hemodynamic and metabolic stresses, glomerular hypertension, chronic hyperglycemia, and activation of pro-inflammatory and pro-fibrotic pathways, culminating in progressive albuminuria and a decline in eGFR. Contemporary standards of care emphasize renin angiotensin aldosterone system (RAAS) blockade with angiotensin-converting enzyme inhibitors (ACEIs) or angiotensin II receptor blockers (ARBs), together with cardiovascular risk reduction and, increasingly, sodium–glucose cotransporter-2 inhibitors (SGLT2i) (1). Yet a substantial “residual risk” of renal progression persists, particularly in patients with established DN and persistent albuminuria despite optimized background therapy. This residual risk has motivated evaluation of mineralocorticoid receptor (MR) antagonism as a complementary, non-glycemic, kidney-protective strategy (2, 3).

Finerenone, a selective, non-steroidal MR antagonist (nsMRA), was designed to attenuate MR-mediated inflammatory and fibrotic signaling in the kidney and heart while limiting off-target endocrine effects characteristic of steroidal MRAs. In large outcome trials, finerenone added to RRAS blockade reduced clinically relevant renal and cardiovascular events and lowered albuminuria across a broad CKD spectrum in type 2 diabetes (T2D) (4). Specifically, FIDELIO-DKD demonstrated a reduction in the primary kidney composite and key cardiovascular composite outcomes, while FIGARO-DKD showed a reduction in major cardiovascular events with supportive effects on kidney outcomes. Prespecified pooled analyses (FIDELITY) reinforced these benefits, including in more advanced CKD, and suggested consistent effects across demographic subgroups (5, 6). Mechanistic analyses further linked early decrements in urinary albumin-to-creatinine ratio (UACR) with subsequent improvements in kidney and cardiovascular outcomes, supporting albuminuria reduction as a mediator of benefit (7–9). Evidence from routine clinical practice is increasingly validating these findings. Recent observational cohorts report UACR reductions and acceptable safety with finerenone in routine practice, and emerging syntheses and meta-analyses over the last three years have consistently indicated kidney and cardiovascular risk reduction, with a modest increase in biochemical hyperkalemia that is generally manageable under standard monitoring protocols. These data align with and extend guideline recommendations from the American Diabetes Association (ADA) Standards of Care 2024 and the KDIGO diabetes-in-CKD guidance, which position finerenone as an add-on to ACEI/ARB in patients with T2D, CKD, and persistent albuminuria, provided eGFR and serum potassium thresholds are met (10, 11).

Although large randomized controlled trials have demonstrated the renoprotective effects of finerenone in patients with diabetic kidney disease, these studies were conducted under highly controlled conditions with stringent inclusion and exclusion criteria, which may limit the applicability of their findings to routine care settings. Patients with DN in clinical practice populations often exhibit heterogeneous disease severity, multiple comorbidities, and variable treatment adherence, all of which may influence therapeutic effectiveness and safety (12, 13). Moreover, evidence regarding the short-term effects of finerenone on dynamic renal function changes, particularly clinically meaningful declines in eGFR, remains limited outside controlled trial environments (14, 15). Therefore, an important evidence gap persists concerning whether finerenone, when added to standard ACEI/ARB therapy, confers independent renal protection without increasing adverse events in patients with DN. To address this gap, the present retrospective cohort study aimed to evaluate the effects of finerenone on renal function parameters and to identify predictors of significant eGFR decline in a clinical practice population.

## 2 Methods

### 2.1 Study design

**Figure 1:**
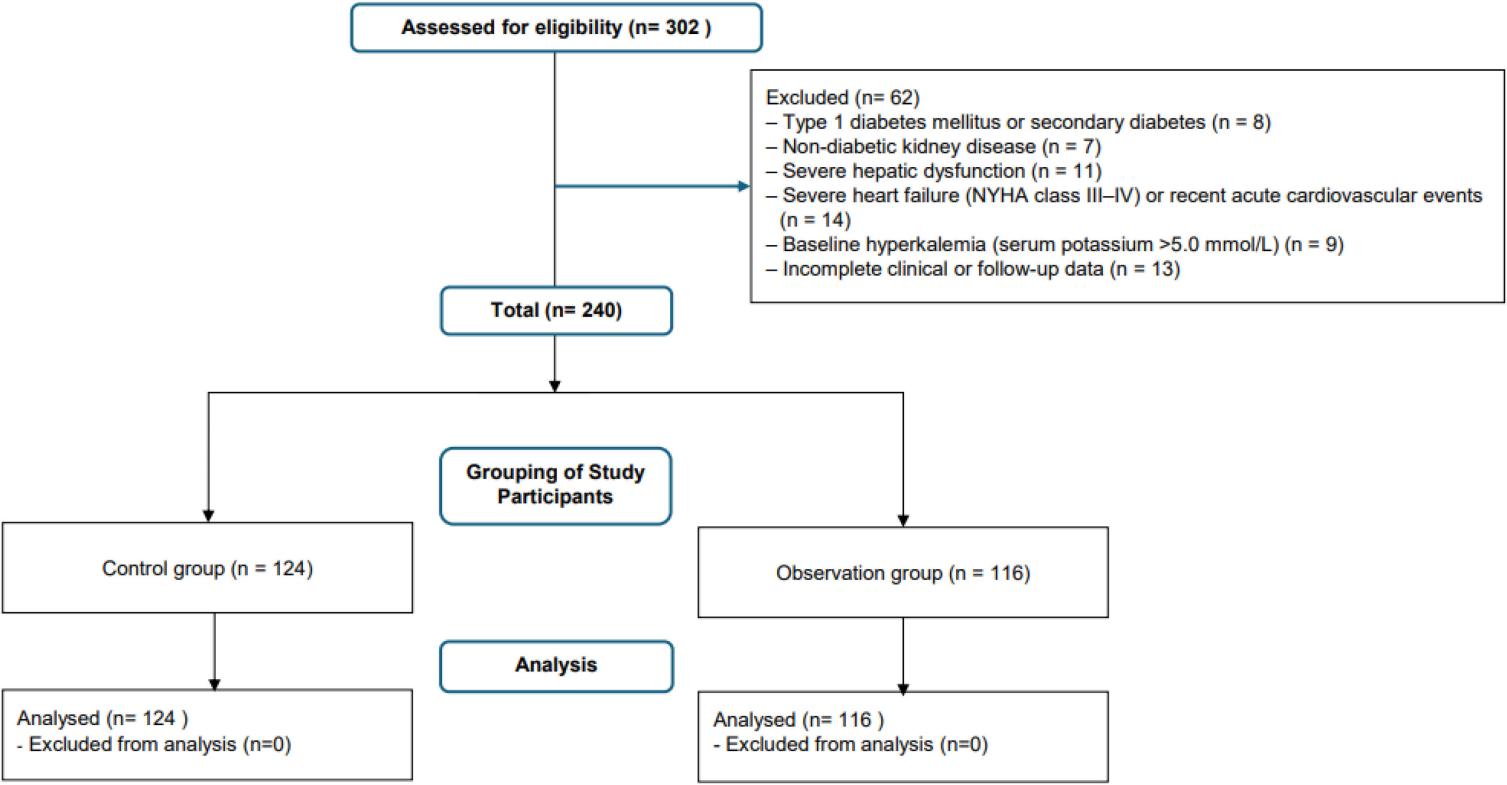
Flowchart of patient selection.

This retrospective cohort study included patients diagnosed with DN who received treatment at our institution between January 2019 and December 2024. Patients treated between January 2019 and December 2022 with ACEIs or ARBs alone were assigned to the control group, while those diagnosed between January 2023 and December 2024 who received combination therapy with ACEI/ARB and finerenone were assigned to the observation group. In this group, finerenone was administered at a fixed dose of 10 mg once daily throughout the treatment period. Patients were eligible for inclusion if they had a confirmed diagnosis of DN based on clinical, laboratory, and imaging findings, in accordance with the diagnostic criteria established by the American Diabetes Association (ADA) (16) and the Kidney Disease: Improving Global Outcomes (KDIGO) guidelines (17); were between 18 and 80 years of age at the time of enrollment; and had complete clinical, biochemical, and follow-up data, including serum creatinine, urinary albumin-to-creatinine ratio (UACR), blood pressure, and glycated hemoglobin (HbA1c). Patients in the observation group were required to have received a stable dose of ACEI/ARB in combination with finerenone for at least 12 weeks, whereas those in the control group received continuous ACEI/ARB monotherapy for the same period. Patients were excluded if they had type 1 diabetes mellitus or secondary diabetes resulting from other endocrine or metabolic disorders, non-diabetic renal disease confirmed by renal biopsy or clinical evaluation, severe hepatic dysfunction (alanine aminotransferase or aspartate aminotransferase levels greater than three times the upper limit of normal), severe heart failure (New York Heart Association Class III–IV) or acute cardiovascular events within the previous three months, or a baseline serum potassium concentration exceeding 5.0 mmol/L or a history of recurrent hyperkalemia (**Figure 1**). Informed consent was obtained from all participants. The study was reviewed and approved by the Ethics Committee of Affiliated Hospital of Hebei University and conducted in accordance with relevant guidelines and the Declaration of Helsinki. All data were anonymized prior to analysis to ensure participant confidentiality.

### 2.2 Diagnostic criteria of diabetic nephropathy

The diagnosis of DN was established according to the criteria jointly recommended by the ADA and KDIGO guidelines. DN was defined as the presence of CKD attributable to diabetes mellitus, characterized by one or more of the following findings persisting for at least three months:

1. Elevated urinary albumin excretion, defined as a urinary albumin-to-creatinine ratio (UACR) ≥ 30 mg/g (3 mg/mmol) in at least two of three consecutive measurements obtained within a six-month period.
2. Reduced renal function, indicated by an eGFR < 60 mL/min/1.73 m², calculated using the CKD-EPI formula.
3. Characteristic clinical and laboratory features, including a long duration of diabetes, persistent albuminuria without other primary renal disease, diabetic retinopathy, and absence of evidence suggesting alternative causes of kidney injury.
4. Exclusion of non-diabetic kidney disease (NDKD) through clinical evaluation, laboratory testing, or renal biopsy when atypical features were present, such as sudden onset of nephrotic-range proteinuria, hematuria with red blood cell casts, rapid decline in eGFR, or absence of diabetic retinopathy.

### 2.3 Data collection

Clinical and laboratory data were retrospectively collected from the electronic medical records of patients diagnosed with DN who received treatment at our institution between January 2019 and December 2024. Demographic, clinical, and biochemical information was obtained at baseline and at the end of the 24-week treatment period. Baseline data included age, sex, duration of diabetes, body mass index (BMI), pathological stage of DN, and chronic kidney disease (CKD) stage. Comorbid conditions such as hypertension, hyperlipidemia, and hyperuricemia were also recorded. Laboratory indicators included serum creatinine (Scr), eGFR, urinary albumin-to-creatinine ratio (UACR), serum potassium, fasting plasma glucose, and glycated hemoglobin (HbA1c). Blood pressure was measured using a standard mercury sphygmomanometer after at least 10 minutes of rest, and the mean of two consecutive readings was used for analysis. Follow-up data were obtained after 24 weeks of continuous therapy. Changes in renal function parameters (Scr, eGFR, and UACR), metabolic indices (HbA1c and serum potassium), and hemodynamic indicators (systolic and diastolic blood pressure) were recorded. The decline rate of eGFR was calculated to evaluate renal function progression, and a ≥15% reduction from baseline was defined as clinically significant deterioration. Adverse events (AEs) were retrospectively identified from patient-reported symptoms and routine clinical documentation in the electronic medical records and were subsequently classified according to severity. Hyperkalemia was categorized as mild (serum K^+^ 5.0–5.5 mmol/L) or moderate-to-severe (K^+^ >5.5 mmol/L). All data were independently verified by two investigators to ensure accuracy and completeness. Missing data were cross-checked with original medical charts, and cases with incomplete key variables were excluded from the final analysis.

### 2.4 Statistical analysis

All statistical analyses were performed using SPSS version 28.0 (IBM Corp., Armonk, NY, USA). Continuous variables were tested for normality using the Kolmogorov–Smirnov test. Normally distributed data were expressed as the mean ± standard deviation (SD), whereas non-normally distributed data were presented as the median (interquartile range, IQR). Between-group comparisons of continuous variables were performed using the independent-samples t test for normally distributed data and the Mann–Whitney U test for non-normally distributed data. Within-group comparisons before and after treatment were conducted using the paired-samples t test or Wilcoxon signed-rank test, as appropriate. Categorical variables were expressed as numbers and percentages [n (%)] and compared between groups using the chi-square (χ²) test or Fisher’s exact test when expected frequencies were <5. The decline rate of eGFR was calculated as the percentage change from baseline to post-treatment. A reduction of ≥15% in eGFR was defined as a clinically significant renal function decline. To identify factors associated with eGFR decline, univariate logistic regression analysis was first conducted for all potential clinical and biochemical predictors. Variables with P < 0.10 in univariate analysis were then entered into a multivariate logistic regression model using the enter method to identify independent predictors. Odds ratios (ORs) and adjusted odds ratios (aORs) with corresponding 95% confidence intervals (CIs) were calculated. All tests were two-sided, and a P value < 0.05 was considered to indicate statistical significance.

## 3 Results

### 3.1 Baseline clinical and pathological characteristics

A total of 240 patients with DN were enrolled in this study, including 124 patients who received ACEI/ARB monotherapy (control group) and 116 patients who received ACEI/ARB in combination with finerenone (observation group). As summarized in **Table 1**, there were no statistically significant differences in baseline demographic or clinical parameters between the two groups, indicating good comparability prior to treatment intervention. The mean age of the patients was 62.4 ± 8.9 years in the control group and 63.1 ± 9.2 years in the observation group, with a predominance of male patients (61.3% and 58.6%, respectively). The mean duration of diabetes and body mass index (BMI) were also comparable between groups (11.3 ± 5.4 years vs. 10.9 ± 5.1 years, and 26.8 ± 3.2 kg/m² vs. 27.1 ± 3.5 kg/m², respectively). In terms of renal pathology, the distribution of DN stages (IIa–IV) was similar in both cohorts (χ² = 3.161, P = 0.368). The majority of patients in both groups were classified as stage IIa or III, reflecting moderate structural kidney injury. Similarly, the chronic kidney disease (CKD) stage distribution (stages 1–4) did not differ significantly between groups (χ² = 2.668, P = 0.114), with most patients presenting with stage 2 or stage 3 CKD, consistent with early-to-mid renal functional decline. Regarding metabolic and cardiovascular comorbidities, the prevalence of hyperlipidemia, hyperuricemia, and hypertension showed no significant differences between the two treatment groups. Hyperlipidemia was observed in 62.1% of patients in the control group and 50.0% in the observation group (P = 0.059), while hyperuricemia was present in 25.8% and 33.6%, respectively (P = 0.185). Hypertension was common in both groups (63.7% vs. 70.7%, P = 0.250). Overall, the baseline characteristics of both groups were well balanced with respect to demographic, clinical, metabolic, and pathological parameters.

**Table 1:** Baseline clinical and pathological characteristics of patients with diabetic nephropathy.

Variable	Control group (n = 124)	Observation group (n = 116)	Test statistic	P value
Age (years)	62.4 ± 8.9	63.1 ± 9.2	t = 0.598	0.550
Male, n (%)	76 (61.3)	68 (58.6)	χ² = 0.178	0.673
Duration of diabetes (years)	11.3 ± 5.4	10.9 ± 5.1	t = 0.590	0.556
Body mass index (kg/m²)	26.8 ± 3.2	27.1 ± 3.5	t = 0.692	0.490
Pathological stage of DN, n (%)			χ² = 3.161	0.368
Stage IIa	50 (40.3)	49 (42.2)		
Stage IIb	16 (12.9)	9 (7.8)		
Stage III	50 (40.3)	45 (38.8)		
Stage IV	8 (6.5)	13 (11.2)		
CKD stage, n (%)			χ² = 2.668	0.114
Stage 1	27 (21.8)	24 (20.7)		
Stage 2	37 (29.8)	43 (37.1)		
Stage 3	47 (37.9)	48 (41.4)		
Stage 4	13 (10.5)	1 (0.9)		
Hyperlipidemia, n (%)	77 (62.1)	58 (50.0)	χ² = 3.564	0.059
Hyperuricemia, n (%)	32 (25.8)	39 (33.6)	χ² = 1.757	0.185
Hypertension, n (%)	79 (63.7)	82 (70.7)	χ² = 1.322	0.250

ACEI, angiotensin-converting enzyme inhibitor; ARB, angiotensin II receptor blocker; DN, diabetic nephropathy; CKD, chronic kidney disease; BMI, body mass index.

### 3.2 Changes in clinical and biochemical parameters after treatment

As shown in **Table 2**, there were no significant differences in baseline indicators between the two groups before treatment. After 24 weeks of therapy, renal function parameters showed significant improvement in the observation group compared with the control group. Serum creatinine was lower (128.3 ± 27.6 vs. 140.8 ± 35.1 μmol/L, P = 0.002), while eGFR was higher (56.8 ± 11.4 vs. 50.1 ± 12.3 mL/min/1.73 m², P < 0.001). UACR also decreased markedly in the finerenone group (301.4 ± 142.7 vs. 398.7 ± 176.8 mg/g, P < 0.001), indicating better renal protection. Serum potassium levels slightly increased in both groups but remained within normal limits, and no significant difference was observed (P = 0.308). The observation group had a greater reduction in systolic blood pressure compared with the control group (P = 0.034), while changes in diastolic blood pressure and HbA1c were not significant.

**Table 2:** Comparison of clinical and biochemical parameters before and after treatment in patients with diabetic nephropathy.

Variable	Time point	Control group (n = 124)	Observation group (n = 116)	t value	P value
Serum creatinine (μmol/L)	Before	136.5 ± 32.4	134.2 ± 29.8	0.573	0.567
	After	140.8 ± 35.1	128.3 ± 27.6	3.077	0.002*
eGFR (mL/min/1.73 m²)	Before	52.8 ± 11.5	53.6 ± 12.1	0.524	0.601
	After	50.1 ± 12.3	56.8 ± 11.4	4.380	<0.001*
UACR (mg/g)	Before	415.6 ± 182.3	428.9 ± 196.5	0.543	0.588
	After	398.7 ± 176.8	301.4 ± 142.7	4.705	<0.001*
Serum potassium (mmol/L)	Before	4.31 ± 0.42	4.35 ± 0.38	0.774	0.439
	After	4.43 ± 0.47	4.49 ± 0.44	1.021	0.308
Systolic BP (mmHg)	Before	138.5 ± 12.7	136.8 ± 13.1	1.020	0.309
	After	133.7 ± 11.9	130.4 ± 12.1	2.128	0.034*
Diastolic BP (mmHg)	Before	82.3 ± 8.6	81.5 ± 9.0	0.703	0.483
	After	79.1 ± 8.2	77.3 ± 8.0	1.721	0.087
HbA1c (%)	Before	7.8 ± 0.9	7.7 ± 0.8	0.911	0.363
	After	7.6 ± 0.8	7.5 ± 0.7	1.032	0.303

Scr, serum creatinine; eGFR, estimated glomerular filtration rate; UACR, urinary albumin-to-creatinine ratio; BP, blood pressure; HbA1c, glycated hemoglobin; SD, standard deviation.

P < 0.05 indicates statistical significance (*).

### 3.3 Risk factors associated with a ≥15% decline in eGFR

To identify factors associated with renal function decline, univariate and multivariate logistic regression analyses were performed, as shown in **Table 3**. In the univariate analysis, longer diabetes duration (OR = 1.074, 95% CI: 1.018–1.133, P = 0.009), lower baseline eGFR (OR = 0.952, 95% CI: 0.925–0.980, P = 0.001), higher baseline UACR (OR = 1.086, 95% CI: 1.027–1.149, P = 0.004), presence of hypertension (OR = 1.752, P = 0.046*), and hyperuricemia (OR = 1.885, P = 0.036*) were significantly correlated with a ≥15% decline in eGFR. In contrast, finerenone therapy was identified as a protective factor against renal deterioration (OR = 0.421, 95% CI: 0.235–0.752, P = 0.004). After adjustment for potential confounders, the multivariate model confirmed that longer diabetes duration (aOR = 1.062, 95% CI: 1.003–1.124, P = 0.039), lower baseline eGFR (aOR = 0.958, 95% CI: 0.930–0.987, P = 0.005), and higher baseline UACR (aOR = 1.072, 95% CI: 1.013–1.135, P = 0.016) were independent predictors of eGFR decline ≥15%. Finerenone treatment remained an independent protective factor (aOR = 0.473, 95% CI: 0.253–0.883, P = 0.019*), suggesting its beneficial effect in slowing kidney function deterioration. Other variables, including age, sex, BMI, blood pressure, and HbA1c, were not significantly associated with eGFR decline in the adjusted model.

**Table 3:** Univariate and multivariate logistic regression analysis of factors associated with ≥15% decline in eGFR.

Variable	Univariate analysis OR (95% CI)	P value	Multivariate analysis aOR (95% CI)	P value
Age (years)	1.018 (0.993–1.043)	0.156	1.012 (0.985–1.040)	0.333
Male sex	1.237 (0.705–2.170)	0.457	1.165 (0.617–2.198)	0.637
Duration of diabetes (years)	1.074 (1.018–1.133)	0.009*	1.062 (1.003–1.124)	0.039*
BMI (kg/m²)	1.052 (0.957–1.158)	0.294	1.048 (0.941–1.167)	0.393
Baseline eGFR (per 1 mL/min/1.73 m²)	0.952 (0.925–0.980)	0.001*	0.958 (0.930–0.987)	0.005*
Baseline UACR (per 100 mg/g)	1.086 (1.027–1.149)	0.004*	1.072 (1.013–1.135)	0.016*
Hypertension	1.752 (1.010–3.038)	0.046*	1.463 (0.796–2.690)	0.217
Hyperlipidemia	1.354 (0.782–2.343)	0.280	1.198 (0.654–2.197)	0.559
Hyperuricemia	1.885 (1.043–3.405)	0.036*	1.712 (0.917–3.196)	0.090
Systolic BP (per 10 mmHg increase)	1.134 (0.947–1.359)	0.174	1.117 (0.915–1.364)	0.272
HbA1c (%)	1.141 (0.879–1.479)	0.326	1.095 (0.827–1.451)	0.534
Finerenone therapy	0.421 (0.235–0.752)	0.004*	0.473 (0.253–0.883)	0.019*

OR, odds ratio; aOR, adjusted odds ratio; CI, confidence interval; BMI, body mass index; eGFR, estimated glomerular filtration rate; UACR, urinary albumin-to-creatinine ratio; BP, blood pressure; HbA1c, glycated hemoglobin.

P < 0.05 indicates statistical significance (*).

### 3.4 Adverse events and safety profile

The incidence and types of adverse events in both groups are summarized in **Table 4**. Overall, both treatment regimens were well tolerated. The total incidence of adverse events was slightly higher in the observation group than in the control group (30.2% vs. 25.0%), but the difference was not statistically significant (χ² = 0.804, P = 0.370). The most common adverse event was mild hyperkalemia (serum potassium 5.0–5.5 mmol/L), which occurred in 8.6% of patients in the observation group and 4.0% of patients in the control group (P = 0.142). Moderate-to-severe hyperkalemia (serum potassium >5.5 mmol/L) was rare and comparable between groups (1.7% vs. 0.8%, P = 0.523). Importantly, no cases of severe or symptomatic hyperkalemia requiring hospitalization or discontinuation were reported. Other adverse events, including dizziness or hypotension, fatigue, gastrointestinal discomfort, and transient elevations in liver enzymes, occurred at low frequencies and showed no significant between-group differences. The incidence of acute kidney injury and treatment discontinuation due to adverse events was also low and similar between the two groups. No serious adverse events (SAEs) were observed during the treatment period. These findings indicate that both treatment regimens were safe and well tolerated. The addition of finerenone to ACEI/ARB therapy did not increase the risk of severe hyperkalemia or other clinically significant adverse effects, consistent with previously reported safety data from large-scale clinical trials.

**Table 4:** Adverse events: between-group comparison.

Adverse event	Control (n=124)	Observation (n=116)	χ² value	P value
Any adverse event, n (%)	31 (25.0)	35 (30.2)	0.804	0.370
Mild hyperkalemia (K^+^ 5.0–5.5 mmol/L), n (%)	5 (4.0)	10 (8.6)	2.154	0.142
Moderate–severe hyperkalemia (K^+^ >5.5 mmol/L), n (%)	1 (0.8)	2 (1.7)	0.409	0.523
Dizziness or hypotension, n (%)	8 (6.5)	6 (5.2)	0.179	0.673
Fatigue, n (%)	9 (7.3)	10 (8.6)	0.153	0.696
Gastrointestinal discomfort, n (%)	6 (4.8)	7 (6.0)	0.167	0.683
Elevated liver enzymes (ALT/AST >2×ULN), n (%)	3 (2.4)	4 (3.4)	0.224	0.636
Acute kidney injury, n (%)	2 (1.6)	1 (0.9)	0.274	0.601
Discontinuation due to AEs, n (%)	2 (1.6)	3 (2.6)	0.278	0.598
Serious adverse events (SAEs), n (%)	0 (0.0)	0 (0.0)	NA	NA

AE, adverse event; ALT, alanine aminotransferase; AST, aspartate aminotransferase; ULN, upper limit of normal; SAEs, serious adverse events.

‘Any adverse event’ denotes the occurrence of at least one adverse event of any type in a given patient during the treatment period.

### 3.5 *Post hoc* power analysis

A *post hoc* power analysis was conducted for the independent predictors retained in the final multivariate logistic regression model, including duration of diabetes, baseline eGFR, baseline UACR, and finerenone therapy. Equal weights were assigned to each predictor to derive a weighted overall estimate of statistical power. Based on the estimated *post hoc* power for each predictor, the weighted *post hoc* statistical power of the model was calculated to be 87.8%, exceeding the conventional adequacy threshold of 80%. These findings indicate that the available sample size provided sufficient statistical power to detect independent associations with a ≥15% decline in eGFR, thereby supporting the robustness and reliability of the multivariate regression results.

## 4 Discussion

This retrospective cohort study evaluated the effectiveness and safety of adding finerenone to background ACEI/ARB therapy in patients with DN. Across a 24-week observation window, combination therapy was associated with lower serum creatinine, higher eGFR, and a greater reduction in albuminuria compared with ACEI/ARB alone, while serum potassium and glycemic control remained stable. Multivariable analyses identified longer diabetes duration, lower baseline eGFR, and higher baseline UACR as independent predictors of an eGFR decline ≥15%, whereas finerenone use independently reduced this risk. Adverse events were infrequent and balanced between groups, with no excess of moderate–severe hyperkalemia. Collectively, these findings indicate that finerenone provides incremental renoprotection beyond optimized renin–angiotensin system blockade without compromising short-term safety (18).

The consistent directionality of the three renal endpoints, serum creatinine, eGFR, and UACR, supports a treatment effect on both glomerular filtration and albuminuria. The absolute between-group difference in eGFR (approximately +6.7 mL/min/1.73 m² in favor of combination therapy at 24 weeks) is clinically relevant at the population level and aligns with the observed albuminuria reduction. Because albuminuria and eGFR change may respond on different time scales, the concordance across these measures strengthens causal inference that finerenone added to ACEI/ARB improves renal physiology rather than merely altering a single surrogate (19). The absence of between-group differences in HbA1c argues against glycemic control as a mediator, and the modest yet significant reduction in systolic blood pressure is unlikely to fully account for the magnitude of UACR improvement, which is consistent with a direct mineralocorticoid receptor (MR)–modulating effect on renal inflammation and fibrosis (20, 21).

The multivariable model provides additional context for risk stratification. Longer diabetes duration, lower baseline eGFR, and higher baseline UACR predicted clinically meaningful eGFR decline. These covariates are well-established markers of progression and function as confounders or effect modifiers in observational cohorts. Their persistence as independent predictors after adjustment indicates that the observed benefit with finerenone is not explained by imbalance in disease severity. The adjusted association of finerenone with a lower odds of ≥15% eGFR decline supports a protective effect in routine practice conditions. Safety signals were acceptable. Mild hyperkalemia occurred more often numerically with finerenone but did not reach statistical significance, and moderate–severe hyperkalemia was rare in both groups (22, 23). The neutral findings for liver enzymes, dizziness/hypotension, and discontinuations are consistent with a favorable short-term tolerability profile when standard potassium monitoring procedures are followed.

Recent guideline and trial syntheses converge on the role of finerenone as an add-on to ACEI/ARB in type 2 diabetes with CKD. The ADA 2024 Standards of Care recommend finerenone for patients with albuminuria and eGFR≥25 mL/min/1.73 m² on maximally tolerated ACEI/ARB, emphasizing benefits on kidney and cardiovascular outcomes and the need for potassium monitoring. Our results are concordant with these recommendations, showing albuminuria reduction and eGFR preservation without an excess of serious hyperkalemia over 24 weeks. Prespecified pooled analyses from FIDELITY (integrating FIDELIO-DKD and FIGARO-DKD) reported that finerenone lowered the risk of kidney disease progression and cardiovascular events across a broad CKD spectrum while increasing hyperkalemia modestly; these analyses include patient-level data and support a class effect beyond single trials. Our cohort demonstrated similar trends in renal parameters and a manageable hyperkalemia profile, consistent with the FIDELITY study, albeit over a shorter time frame and in a clinical practice setting (22, 24, 25).

A 2024–2025 stream of evidence, including narrative syntheses and meta-analyses, has reinforced the dual kidney–cardiovascular benefit of finerenone and clarified its safety relative to steroidal MRAs (26). And recent reviews highlight finerenone’s non-steroidal selectivity, reduced off-target endocrine effects, and lower risk of worsening kidney function compared with spironolactone, framing a rationale for broader adoption in CKD with diabetes (27, 28). These sources report sustained UACR reductions and attenuation of eGFR decline, congruent with our findings. Notably, pooled data and meta-analyses confirm a higher relative risk of biochemical hyperkalemia versus control, but with low absolute event rates and infrequent clinical sequelae when monitored. The absence of severe hyperkalemia and the low discontinuation rate in our cohort are consistent with this risk–benefit profile (29). From a guideline perspective, KDIGO’s 2022 diabetes in CKD update and its quick reference recommend comprehensive kidney-protective therapy combining ACEI/ARB, SGLT2 inhibition, and finerenone in appropriate patients. Although our dataset did not stratify by SGLT2 inhibitor use, the observed renal benefits with finerenone on top of ACEI/ARB align with KDIGO’s integrated approach and suggest that further gains may be achievable with triple therapy in eligible individuals.

Finerenone’s selective, non-steroidal MR antagonism reduces pro-inflammatory and pro-fibrotic signaling in the kidney, including in mesangial and tubular compartments. The quantitatively greater effect on UACR compared with systolic blood pressure suggests that antiproteinuric actions are not solely hemodynamic. The stability of HbA1c argues that the renal signal is independent of glycemic modulation. Together, these elements are consistent with the hypothesized pathway in which MR overactivation promotes renal inflammation and fibrosis; antagonism attenuates albuminuria and slows eGFR loss beyond blood pressure effects. Emerging translational work also indicates potential benefits in cardiorenal crosstalk, but our dataset was not powered for cardiovascular endpoints (30, 31).

The present findings support adding finerenone to maximally tolerated ACEI/ARB in adults with DN and persistent albuminuria, particularly in patients with longer diabetes duration, lower baseline eGFR, or higher UACR who are at increased risk for progression. Routine potassium monitoring enables early identification and management of mild hyperkalemia without compromising therapy. The absence of glycemic or substantial hemodynamic confounding suggests that finerenone can be layered onto stable diabetes and hypertension regimens. These data are aligned with current ADA and KDIGO guidance and can inform institutions seeking to standardize cardiorenal-protective pharmacotherapy pathways in diabetes-associated CKD. Strengths include a relatively large single-center cohort with balanced baseline characteristics and harmonized outcome collection at prespecified time points. The analysis incorporated both creatinine-based filtration and albuminuria, enabling triangulation across complementary renal endpoints. Use of multivariable logistic regression identified independent predictors of clinically meaningful eGFR decline and quantified the association between finerenone exposure and progression risk, enhancing interpretability for practice.

Several strengths of this study merit consideration in the context of existing literature and support its clinical relevance. Unlike large randomized controlled trials conducted under highly controlled conditions, the present cohort represents a heterogeneous population of patients with DN encountered in routine clinical practice, thereby enhancing the applicability of the findings to everyday care. Renal outcomes were comprehensively assessed using complementary indices, including serum creatinine, eGFR, and UACR, rather than relying on a single surrogate marker, allowing for a more robust and multidimensional evaluation of renal function changes. Importantly, the study focused on a clinically meaningful endpoint—an eGFR decline of ≥15%, which captures early renal deterioration relevant to disease progression and therapeutic decision-making. In addition, multivariable logistic regression was applied to adjust for potential confounders and to identify independent predictors of renal function decline, enabling risk stratification of patients who may derive greater benefit from finerenone therapy. The predefined follow-up duration and standardized data collection across time points further ensured consistency in outcome assessment and strengthened the internal validity of the analysis. From a clinical perspective, the present findings suggest that the addition of finerenone to ACEI/ARB therapy confers independent renal protection, as evidenced by significant improvements in serum creatinine, eGFR, and UACR over a 24-week period. These results are concordant with current clinical guidelines, including the ADA 2024 Standards of Care and KDIGO recommendations, which endorse finerenone as an adjunctive therapy in patients with albuminuria and an eGFR ≥25 mL/min/1.73 m². The study further demonstrates that finerenone can be administered safely with routine potassium monitoring, without an increased risk of severe hyperkalemia, consistent with the established safety profile observed in large outcome trials such as FIDELITY. Clinically, these data support the integration of finerenone into standard care pathways for patients with type 2 diabetes and DN, particularly those at higher risk of renal progression due to longer diabetes duration, lower baseline eGFR, or higher baseline UACR. The manageable hyperkalemia profile observed underscores the feasibility of this strategy in routine practice, where appropriate monitoring and early intervention can mitigate potential risks. Collectively, the findings reinforce the role of finerenone in optimizing multimodal renal and cardiovascular protection in DN.

Several limitations should be acknowledged. First, the retrospective observational design introduces potential selection bias and residual confounding, and causal inference remains limited despite multivariable adjustment. Important factors such as medication adherence, dietary potassium intake, and concurrent nephrotoxic exposures were not systematically captured and may have influenced treatment effects. Second, the follow up duration was relatively short at 24 weeks, which precludes assessment of long term renal and cardiovascular outcomes, including progression to end stage kidney disease or mortality, as well as the durability of renoprotective benefits. Third, data on concomitant use of other kidney protective therapies, particularly sodium glucose cotransporter 2 inhibitors and glucagon like peptide 1 receptor agonists, were not stratified, limiting evaluation of potential additive or synergistic effects. Fourth, ascertainment of hyperkalemia relied on routine clinical laboratory testing rather than protocolized frequent monitoring, which may underestimate transient biochemical events. Fifth, renal function assessment was based on creatinine derived eGFR and albuminuria without serial cystatin C measurements or hard renal endpoints, which may fail to capture subtle or early changes in kidney function. Future research should therefore prioritize prospective cohorts or pragmatic trials with longer follow up to validate these findings and to examine long term renal and cardiovascular outcomes. Studies incorporating standardized reporting of background therapies, detailed adherence assessment, and expanded biomarker evaluation may further refine patient selection and monitoring strategies, thereby clarifying the optimal integration of finerenone into comprehensive cardiorenal protective treatment pathways.

## 5 Conclusions

In patients with DN, adding finerenone to ACEI/ARB therapy significantly improved renal function, as reflected by lower serum creatinine, improving eGFR, and reduced albuminuria, without increasing the risk of hyperkalemia or other adverse events. Longer diabetes duration, lower baseline eGFR, and higher UACR were associated with renal function decline, whereas finerenone independently conferred renal protection and was well tolerated.

## Data Availability

The raw data supporting the conclusions of this article will be made available by the authors, without undue reservation.
